# 2063. Implementation of a Rapid HIV Screening Program in the Emergency Department

**DOI:** 10.1093/ofid/ofac492.1685

**Published:** 2022-12-15

**Authors:** Matthew Geringer, Brooke A Lajeunesse, Caitlin Bettger, Elizabeth Markelz, Miguel A Arroyo Cazurro, Wesley E Trueblood, Jason F Okulicz

**Affiliations:** Brooke Army Medical Center / San Antonio Uniformed Services Health Education Consortium, San Antonio, Texas; San Antonio Uniformed Services Health Education Consortium, Converse, Texas; Brooke Army Medical Center, Fort Sam Houston, Texas; Brooke Army Medical Center, Fort Sam Houston, Texas; Brooke Army Medical Center, Fort Sam Houston, Texas; Brooke Army Medical Center, Fort Sam Houston, Texas; San Antonio Military Medical Center, San Antonio, Texas

## Abstract

**Background:**

Guidelines recommend that human immunodeficiency virus (HIV) screening be performed for all patients evaluated for sexually transmitted infections (STIs). The current practice for STI evaluation in the Brooke Army Medical Center (BAMC) emergency department (ED) is to defer HIV testing to Primary Care Managers (PCMs), however PCM follow-up and HIV screening may not occur. This project evaluated HIV screening practices before and after implementation of rapid HIV testing in the ED.

**Methods:**

The pre-intervention period (Aug – Oct 2021) included usual practice in the BAMC ED followed by the post-intervention period (Dec 2021 – Feb 2022) after implementation of rapid testing with the Determine™ HIV-1/2 Ag/Ab Combo test. ED providers were educated to include HIV rapid testing for patients with STI complaints. Patients with *Neisseria gonorrhea/Chlamydia trachomatis* (GC/CT) tests ordered in the ED pre-intervention (n=303) and post-intervention (n=268) were selected for chart review and demographic, clinical, and laboratory data were used to assess HIV screening practices.

**Results:**

A similar proportion of patients in the pre-intervention period presented with an STI chief complaint (13.5% vs. 17.2%), tested positive for GC/CT (13.5% vs. 10.8%), and received empiric treatment for GC/CT (38.3% vs. 34.3%) compared to the post-intervention period (Table 1). HIV screening in the ED significantly increased both overall (4.3% vs. 19.8%; P< 0.001) and in the subgroup treated empirically for GC/CT (9.7% vs. 30.4%; P< 0.001, Table 2). Among patients treated empirically for GC/CT who did not receive HIV screening in the ED, PCM follow-up was low in both the pre- and post-intervention periods (20.7% and 34%, respectively; P< 0.001) and HIV screening was not commonly performed by PCMs during those visits (6.2% vs. 8.4%, respectively; P=0.350).

**Figure:**
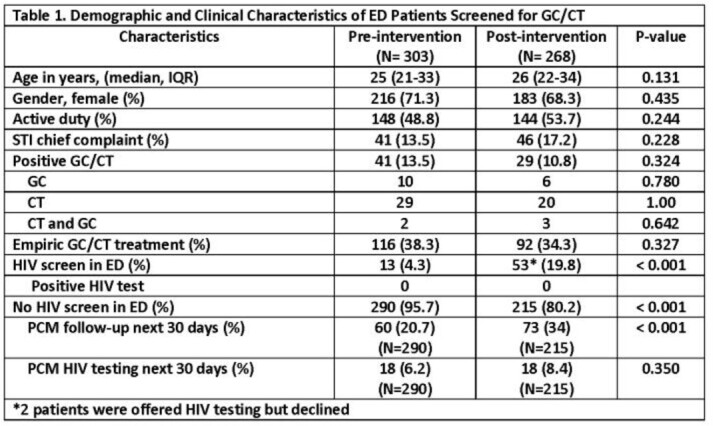

**Figure:**
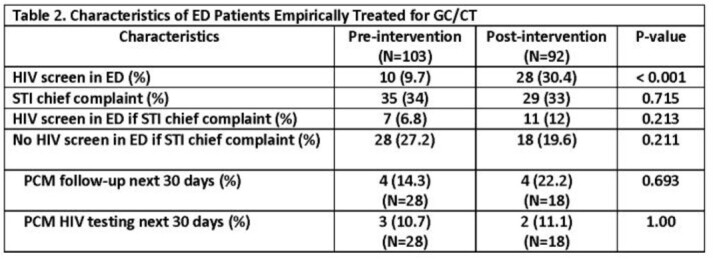

**Conclusion:**

STIs are considered biologic markers of HIV risk, including acquisition and forward transmission. Implementation of a rapid screening protocol in the ED resulted in a nearly 5-fold increase in HIV screening, however HIV screening by PCMs remained low. Although rapid HIV testing can be a useful tool, continued education and training of ED providers and PCMs is also needed to improve uptake of HIV screening.

**Disclosures:**

**All Authors**: No reported disclosures.

